# Telemental Health For Youth With Chronic Illnesses: Systematic Review

**DOI:** 10.2196/30098

**Published:** 2021-08-27

**Authors:** Nancy Lau, Susannah F Colt, Shayna Waldbaum, Alison O'Daffer, Kaitlyn Fladeboe, Joyce P Yi-Frazier, Elizabeth McCauley, Abby R Rosenberg

**Affiliations:** 1 Palliative Care and Resilience Lab Center for Clinical and Translational Research Seattle Children’s Research Institute Seattle, WA United States; 2 Department of Psychiatry and Behavioral Sciences University of Washington School of Medicine Seattle, WA United States; 3 Cambia Palliative Care Center of Excellence University of Washington Seattle, WA United States; 4 Perelman School of Medicine University of Pennsylvania Philadelphia, PA United States; 5 Chicago Medical School Rosalind Franklin University of Medicine and Science North Chicago, IL United States; 6 Department of Pediatrics University of Washington School of Medicine Seattle, WA United States; 7 Center for Child Health, Behavior, and Development Seattle Children’s Research Institute Seattle, WA United States

**Keywords:** telehealth care, mental health, psychosocial issues, psychiatry, psychology, child, chronic disease

## Abstract

**Background:**

Children, adolescents, and young adults with chronic conditions experience difficulties coping with disease-related stressors, comorbid mental health problems, and decreased quality of life. The COVID-19 pandemic has led to a global mental health crisis, and telemental health has necessarily displaced in-person care. However, it remains unknown whether such remote interventions are feasible or efficacious. We aimed to fill this research-practice gap.

**Objective:**

In this systematic review, we present a synthesis of studies examining the feasibility and efficacy of telemental health interventions for youth aged ≤25 years with chronic illnesses.

**Methods:**

PubMed, Embase, Web of Science, PsycInfo, and Cochrane Database of Systematic Reviews were searched from 2008 to 2020. We included experimental, quasiexperimental, and observational studies of telemental health interventions designed for children, adolescents, and young adults aged ≤25 years with chronic illnesses, in which feasibility or efficacy outcomes were measured. Only English-language publications in peer-reviewed journals were included. We excluded studies of interventions for caregivers or health care providers, mental health problems not in the context of a chronic illness, disease and medication management, and prevention programs for healthy individuals.

**Results:**

We screened 2154 unique study records and 109 relevant full-text articles. Twelve studies met the inclusion criteria, and they represented seven unique telemental health interventions. Five of the studies included feasibility outcomes and seven included efficacy outcomes. All but two studies were pilot studies with relatively small sample sizes. Most interventions were based on cognitive behavioral therapy and problem-solving therapy. The subset of studies examining intervention feasibility concluded that telemental health interventions were appropriate, acceptable, and satisfactory to patients and their parents. Technology did not create barriers in access to care. For the subset of efficacy studies, evidence in support of the efficacy of telemental health was mixed. Significant heterogeneity in treatment type, medical diagnoses, and outcomes precluded a meta-analysis.

**Conclusions:**

The state of the science for telemental health interventions designed for youth with chronic illnesses is in a nascent stage. Early evidence supports the feasibility of telehealth-based delivery of traditional in-person interventions. Few studies have assessed efficacy, and current findings are mixed. Future research should continue to evaluate whether telemental health may serve as a sustainable alternative to in-person care after the COVID pandemic.

## Introduction

Children, adolescents, and young adults with chronic medical conditions experience difficulties coping with disease-related stressors and decreased quality of life [1-3]. There is a strong association between physical and mental health in children, adolescents, and young adults, such that up to 60% of those with chronic illnesses are diagnosed with comorbid mental health disorders [4,5]. Common challenges include navigating diagnosis- and treatment-related distress, disruptions to normative development, changing family and peer relationships, and worries and uncertainty about the future [4-6]. The global COVID-19 pandemic has compounded these challenges and led to an increased risk of mental health symptoms, such as anxiety, depression, substance abuse, and posttraumatic stress, in healthy populations and worsening symptoms in those with pre-existing mental health disorders [7]. Further exacerbating negative impacts on psychological health, school closures have resulted in a lack of access to nonacademic support services (eg, sports and extracurricular programs and school mental health counselors), adjustment problems, and social isolation [8-10].

Overburdened health care systems have experienced a corresponding increase in demand for mental health services, and over 200 affected countries have inadequate resources to meet this influx [11,12]. Since the early stages of the pandemic, social distancing precautions have necessitated a shift in the mental health treatment standard of care from an in-person mode of delivery to telemental health (ie, via secure web-based videoconferencing platforms) [7]. Payor policy changes have been implemented to provide much needed care while ensuring the physical safety of patients and providers alike [13]. Projections suggest that US $250 billion of health care spending in the United States could become telehealth-based after the COVID-19 pandemic [14].

The rapid adoption of telehealth in health care systems and insurance program coverage has helped ensure continuity in mental health care and availability of psychosocial services in response to escalating needs during the pandemic. A number of systematic reviews in adult populations have shown that videoconferencing psychotherapies are feasible and have comparable outcomes to in-person treatment, and for anxiety and depression in particular [15-17]. There is limited information regarding the feasibility and efficacy of telemental health services for children, adolescents, and young adults with chronic illnesses, but early findings are similarly promising to adult interventions. Existing pediatric telemental health research has been limited to case studies, single-site child psychiatry department implementation efforts, and reviews pertaining to the treatment of youth with mental health concerns not in the context of medical conditions [18-22].

In this systematic review, we aimed to answer the following research questions:

1. What is the evidence for the feasibility of telemental health in child, adolescent, and young adult chronic illness populations?
2. What is the evidence for the efficacy of telemental health in child, adolescent, and young adult chronic illness populations?
3. What types of in-person interventions and intervention components have been adapted to telemental health delivery?

## Methods

### Data Sources

An electronic database search was executed by a research librarian in five databases (PubMed/MEDLINE, Embase, Web of Science, PsycInfo, and Cochrane Database of Systematic Reviews) on May 29, 2020, for publications from 2008 to 2020. The list of keyword parameters was based on controlled vocabulary terms prespecified by each database. The search terms included those relevant to age demographics, telemedicine and telehealth, and chronic disease, utilizing the following Boolean [23] operators: (p?ediatric* OR child* OR youth* OR teen* OR preteen* OR preadolescent* OR adolescent* OR young adult*) AND (telehealth OR eHealth OR telemedicine OR online OR telecounsel* OR teletherapy*) AND (chronic* OR condition* OR disease* OR ill* OR sick* OR syndrom* OR chronic condition OR chronic disease OR chronic illness OR long term condition OR long term disease OR noncommunicable disease OR noncommunicable condition).

### Study Selection

The inclusion criteria were as follows: (1) availability in English; (2) study published in a peer-reviewed journal; (3) experimental, quasiexperimental, or observational study in which feasibility and/or efficacy outcomes were reported; (4) telemental health interventions delivered via videoconferencing platforms; and (5) interventions designed for children, adolescents, or young adults aged ≤25 years with a chronic disease (ie, a long-term medical condition lasting 3 months or longer [24]). We excluded studies of interventions that targeted caregivers or health care providers only, interventions that targeted mental health problems not in the context of a chronic illness, prevention programs for healthy individuals, and programs that targeted disease and medication management. In addition, we excluded nonpeer-reviewed publications (eg, dissertation manuscripts and conference abstracts) and study protocols for which no outcomes were reported.

First, we screened the titles and abstracts of studies retrieved for inclusion and exclusion. We then obtained the full texts of articles designated as potentially meeting the inclusion criteria to assess eligibility. Screening of all titles, abstracts, and full-text articles was first conducted by two independent coders (NL and SW). Then, disagreements between the authors were discussed while referencing the original source material to reach consensus. For screening of titles and abstracts, interrater agreement between independent coders (NL and SW) was very good, reflected by a Cohen kappa of 0.84. For screening of full-text articles, interrater agreement between independent coders (NL and SW) was good, reflected by a Cohen kappa of 0.79. For articles meeting the inclusion criteria, we independently double coded relevant information from each study in pairs from a group of three (NL, SFC, and KF).

### Data Extraction and Synthesis

Data were extracted using a shared Excel template. Relevant information from each study included study design, sample size, target illness, participant age range, type of control group (where applicable), intervention name, intervention type, facilitator credentials, parental involvement, 1:1 or group-based format, homework assignments, technological components, and results. Both significant and nonsignificant outcomes were reported; we included information on *P* values and effect sizes if reported in the original study publication. After review of the articles meeting the inclusion criteria (n=12), the team determined that heterogeneity in intervention type, study design, outcome variables, and measurement timepoints precluded a meta-analysis. Thus, we described the data in a narrative synthesis.

### Quality Assessment

For the subset of included records that were efficacy studies, study quality was assessed by two independent coders from a group of three (NL, SFC, and KF) using the Cochrane Collaboration tool for assessing risk of bias [25]. The Cochrane tool evaluates the following seven evidence-based categories: random sequence generation (selection bias), allocation concealment (selection bias), blinding of participants and personnel (performance bias), blinding of outcome assessment (detection bias), incomplete outcome data (attrition bias), selective reporting (reporting bias), and other bias. We coded each category as low, high, or unclear risk of bias according to established standards in the *Cochrane Handbook for Systematic Reviews of Interventions* [26]. Support for judgment was directly quoted from the articles or published study protocols (where available) as relevant source materials. We utilized the well-established Grades of Recommendation, Assessment, Development, and Evaluation (GRADE) approach to evaluate the quality of evidence [27]. According to this approach, randomized trials start as high quality and are downgraded for limitations such as lack of allocation concealment; lack of blinding; attrition bias due to amount, nature, or handling of incomplete outcome data; and reporting bias. We coded a category as unclear if not enough information was available in the article to make a judgment. We resolved discrepancies in coding during regularly scheduled consensus meetings by referring back to the journal articles themselves.

## Results

### Overview

**Figure 1:**
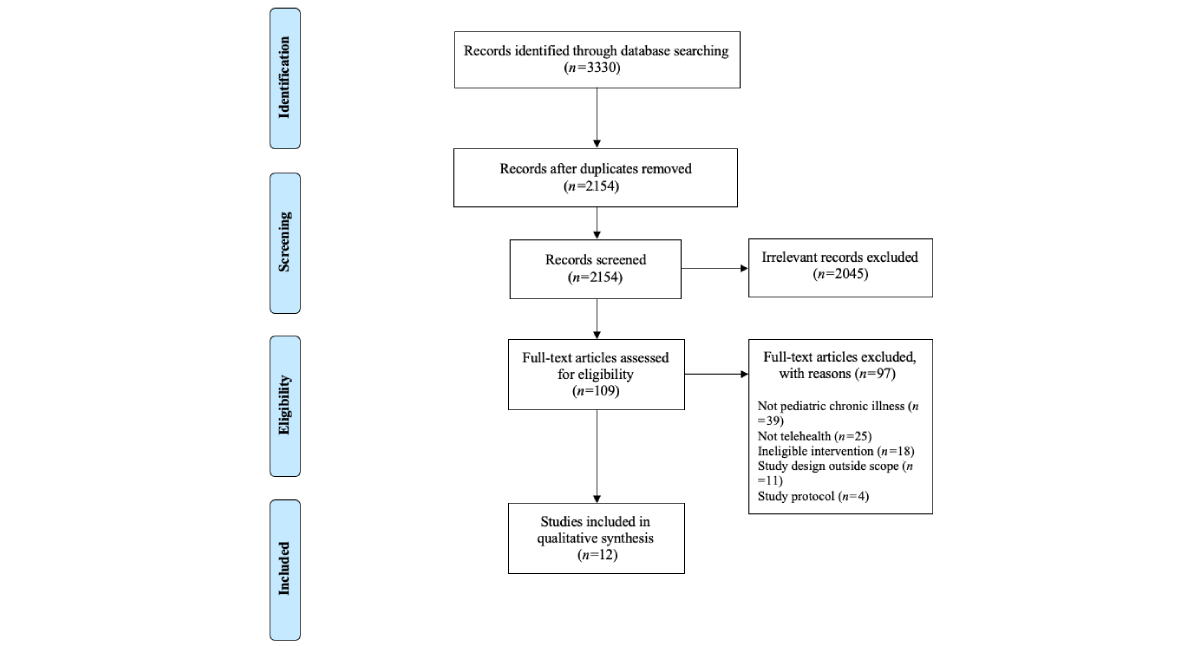
PRISMA (Preferred Reporting Items for Systematic Reviews and Meta-Analyses) flow diagram.

The search initially identified a total of 3330 articles (PubMed, 1410; Embase, 838; Web of Science, 390; PsycInfo, 607; and Cochrane Database of Systematic Reviews, 85). There were 1176 duplicates removed. This resulted in 2154 unique records screened, and 109 full-text articles were designated as potentially meeting the inclusion criteria. Review of the full-text articles resulted in 12 articles that met the criteria for inclusion. The results of the search and selection of studies are described in the PRISMA (Preferred Reporting Items for Systematic Reviews and Meta-Analyses) flow diagram (Figure 1).

### Intervention Characteristics

We found seven unique telemental health interventions that were developed and tested in Australia, Canada, and the United States (Tables 1 and 2). Five (71%) were 1:1 interventions and the rest were group-based interventions. For group-based interventions, the number of facilitators ranged from 1 to 2 and the number of patients ranged from 3 to 9 per group. Four (57%) of the interventions were delivered by psychologists; other facilitators included study personnel with training in the tested intervention (n=2) and therapists at a master’s level (n=1).

**Table 1:** Videoconferencing interventions targeted for youth chronic illness populations.

Source, year	Intervention name	Target illness	Age range, years	Country of origin	Underlying intervention theory	Facilitator credentials	Group based	Facilitator-to-patient ratio
Wade et al, 2020 [28] Moscato et al, 2019 [29]	A Survivor’s Journey^a^	AYA^b^ pediatric brain tumor survivors	13-24^c^	United States	Cognitive-behavioral problem-solving therapy	Licensed clinical psychologist	No	1:1
Wade et al, 2014 [30] Wade et al, 2015 [31]	Counselor Assisted Problem-Solving (CAPS) intervention	Adolescents with traumatic brain injury	12-17	United States	Family-based problem-solving therapy	Licensed clinical psychologist	Yes	1:1
Chadi et al, 2018 [32] Chadi et al, 2019 [33]	Mindful Awareness and Resilience Skills for Adolescents (MARS-A) Program^d^	Chronic medical or mental health illness	13-18	Canada	Evidence-based mindfulness	Experienced personnel with MARS-A specific training	Yes	2:9
Mcgill et al, 2017 [34] Sansom-Daly et al, 2019 [35]	Recapture Life	AYA cancer survivors	15-25	Australia	Cognitive behavioral therapy	Psychologist	Yes	1:3-5
Wade et al, 2018 [36]	Social Participation and Navigation (SPAN) Program	Adolescents with acquired brain injury	14-22	United States	Social skills, problem-solving therapy, goal setting	Trained undergraduate college student coach	No	1:1
Wade et al, 2011 [37]	Teen Online Problem-Solving (TOPS) Intervention	Adolescents with traumatic brain injury	11-18	United States	Family-based problem-solving therapy	Licensed clinical psychologist and clinical psychology PhD students	No	1:1
Ricketts et al, 2016 [38]	VoIP-delivered CBIT (CBIT-VoIP)	Youth with chronic tic disorders	8-16	United States	Comprehensive behavioral intervention for tics (CBIT)	CBIT trained therapist, master's level	No	1:1

^a^A Survivor’s Journey information represents articles by Moscato et al [29] and Wade et al [28], which use the same study to report on different outcomes.

^b^AYA: adolescent and young adult.

^c^This is the age range of the study sample used in analysis. The inclusion criteria specified an age range of 13-25.

^d^MARS-A program information represents articles by Chadi et al [32,33], which are two studies of the same intervention.

**Table 2:** Technology, intervention, and group components.

Source, year	Intervention name	Technology components			Intervention components		
		Video platform used	Provided device	Tech support	Parents involved	Homework assignments	Self-guided
Wade et al, 2020 [28] Moscato et al, 2019 [29]	A Survivor’s Journey^a^	Skype	Yes^b^	Yes^c^	No	Yes	Yes
Wade et al, 2014 [30] Wade et al, 2015 [31]	CAPS intervention	Skype	Yes	Yes^c^	Yes	Yes	Yes
Chadi et al, 2018 [32] Chadi et al, 2019 [33]	Mindful Awareness and Resilience Skills for Adolescents (MARS-A) Program^d^	Zoom	No	Yes	No	Yes	No
McGill et al, 2017 [34] Sansom-Daly et al, 2019 [35]	Recapture Life	WebEx	Yes^b^	No	Yes	Yes	No
Wade et al, 2018 [36]	Social Participation and Navigation (SPAN) Program	Skype	No	No	No	Yes	Yes
Wade et al, 2011 [37]	Teen Online Problem Solving (TOPS) intervention	Not specified	Yes^b,e^	Yes^c^	Yes	Yes	Yes
Ricketts et al, 2016 [38]	VoIP-delivered CBIT (CBIT-VoIP)	Skype	Yes^b^	Yes	Yes	Yes	No

^a^A Survivor’s Journey information represents articles by Moscato et al [29] and Wade et al [28], which report on different outcomes from the same parent study.

^b^Provided to those who did not have one of their own.

^c^Tutorial provided before the start of the intervention; no ongoing tech support.

^d^MARS-A program information represents articles by Chadi et al [32,33], which are two studies of the same intervention.

^e^High speed internet access provided to everyone.

All psychosocial interventions were adapted specifically for chronic medical conditions and designed to teach coping skills to facilitate adjustment to illness via treatment manuals. Interventions were primarily based on evidence-based cognitive behavioral therapy and problem-solving therapy. The specific coping skills targeted included cognitive restructuring, mindfulness, behavioral activation, social participation, goal setting, and problem solving to facilitate adjustment to illness. Manualized intervention content (ie, standardized treatment manuals documenting session-by-session objectives and procedures to ensure intervention fidelity across facilitators) provided a systematic approach for developing adaptive coping strategies and is the standard of practice for empirically supported psychotherapies. All interventions assigned homework to facilitate skills practice. Four (57%) interventions contained self-guided online modules with disease-relevant resources in addition to regularly scheduled videoconference-based therapy sessions. For the subset of interventions with web content, participants met with a facilitator for weekly telemental health sessions. Online self-guided modules consisted of interactive didactic content, videos of patients discussing use of coping skills, and animated videos providing examples of how to directly apply coping skills to day-to-day life. Parents were active participants in family-based videoconferencing therapy sessions for 4 (57%) of the interventions.

The video platforms used were Skype (n=4), Cisco WebEx (n=1), Zoom (n=1), and unspecified (n=1). Five (71%) of the interventions provided devices to connect to video platforms for participants who needed them, and one intervention provided both devices and high-speed internet access for participants. Three (43%) of the interventions provided an introductory tutorial on how to access and use the video platform program, and 2 (29%) provided ongoing technological support.

### Participants

Across all included studies, study sample sizes ranged from 14 to 132. The age range of participants was from 8 to 25 years. The targeted chronic illnesses included brain tumor, cancer, traumatic brain injury, chronic tic disorder, and chronic illness (nondisease-specific intervention). The key study characteristics are summarized in Tables 3 and 4.

**Table 3:** Original research publications reporting on feasibility outcomes.

Source, year	Intervention name	Study type	Sample size^a^	Age range, years	How constructs were defined and measured	Results
Moscato et al, 2019 [29]	A Survivor’s Journey	Pilot feasibility study^b^	17	13-24	Feasibility: Enrollment and completion rates Acceptability: Internally developed satisfaction survey of the Teen Online Problem Solving (TOPS) intervention on a 4-point Likert scale. Overall satisfaction ratings included whether the program met expectations, what content was most and least helpful, whether the website was easy to use, understand, and navigate. System Usability Scale (5-point Likert scale) was used to measure ease of use.	Feasibility: 50% enrollment rate (which met researchers’ enrollment aim), and 95% completed core sessions, which exceeded the goal of a 75% completion rate. Acceptability: Exceeded the goal of 75% of participants reporting satisfaction on most items of the satisfaction survey (eg, every participant reported that they would recommend the program to others, website was easy to use and navigate, and content was relevant to them). Did not meet the goal of 75% of participants rating the intervention above average in usability on the System Usability Scale.
Chadi et al, 2018 [32]	Mindful Awareness and Resilience Skills for Adolescents (MARS-A) Program	Qualitative portion of randomized mixed methods trial	18	13-18	Acceptability/feasibility: Program exit interviews used to foster personal reflections about participants’ experiences of the MARS-A program and qualitative analysis identified four themes from interview data.	Acceptability/feasibility: Themes describing experiences for both in-person and eHealth groups were as follows: creating a safe space; fostering peer support and connection; integrating mindfulness skills into daily life; and improving well-being through mindfulness. Based on qualitative results, they concluded that eHealth delivery of a mindfulness-based intervention may be an acceptable and feasible mode of delivery for adolescents with chronic illnesses.
McGill et al, 2017 [34]	Recapture Life	Qualitative analysis of three-arm pilot randomized controlled trial (RCT)	39^d^	15-25	Acceptability: Authors stated that therapeutic alliance (collaborative element of the patient-therapist dyad) and group cohesion (quality of interpersonal processes between group members and between group members and the therapist) are important to determine the acceptability of online models of psychosocial care.	Acceptability-group cohesion: All participants endorsed at least moderate group cohesion on all group cohesion items after the last session (that they shared important things, felt accepted and respected, and the program was the best way to get help, and it helped them gain a deeper understanding). Acceptability-therapeutic alliance: All participants endorsed strong therapeutic alliance on all therapeutic alliance items, and it remained high over time from the first to last session (understanding, confidence, appreciation, and working correctly). Therapists endorsed strong therapeutic alliance on all therapeutic alliance items (participant comfort, rapport, openness, trust, peer to peer, motivation, and engagement); endorsed that participants had significantly increased openness, trust, and motivation from the first to last session (*P*<.05); and endorsed that the items pertaining to participant comfort, rapport, peer-to-peer discussion, and engagement were strong and unchanged over time from the first to last session.
Sansom-Daly et al, 2019 [35]^c^	Recapture Life	Three-arm RCT reporting on feasibility and acceptability	45^d^	15-25	Feasibility: Recruitment rates across sites; mean days to group commencement; median time for session commencement; proportion of eligible, interested adolescents and young adults (AYAs) who had the technological equipment and internet access required to participate; number and type of technological difficulties experienced across sessions and perceived impact on content delivery; time taken to check participants’ between-session emotional safety using email/text inquiries; total number of additional catch up sessions conducted for AYAs who missed their group session, rescheduled group sessions, and scheduled group sessions outside of office hours. Acceptability: Opt-in, enrollment, and retention rates, and participant engagement (total group sessions attended) and homework completion rates. Responded to two internally developed items “Was participation in this study beneficial to you in any way?” and “Was participation in this study burdensome for you in any way?” Qualitative analysis of open-ended questionnaire responses used to explore participants’ experiences with the program.	Results: Recruitment rate of 30.41% (45 randomized/148 approached), and 80% of participants had access to all required technologies. Individuals waited on average for 40 days (range 5-107) from completing the baseline questionnaire to commencing an online group with a sufficient number of peers. Sessions took a median of 4 minutes to commence, and 74% of sessions had all participants log on within 5 minutes of the scheduled start time. Six “catch up” sessions were delivered for participants unable to attend the scheduled group. Overall, 10 of 12 groups required sessions to be scheduled out-of-hours, representing 60 online sessions across the trial (approximately 90 hours). Technological difficulties were common, being experienced at least once in 71% of sessions, and 38% experienced two or more technological difficulties, but difficulties were rated as having a low impact on intervention delivery. The most common technological difficulties were poor quality audio and dropouts (43% of sessions) and webcam freezing (43% of sessions). Of participants whose scores triggered a between-session telephone call for safety, all were telephoned within 48 hours. An average of 1.8 (range 1-4) email, text, and/or phone calls was required to confirm safety. Authors concluded that the findings support program feasibility. Acceptability: Opt-in rate of 30%. Enrollment rate of 87% of those who completed baseline, and completion rate of 92%. High level of engagement with majority of participants attending at least 74% (5/6) of sessions. AYAs reported a high benefit and low burden of participation on open-ended questionnaire responses. Participants reported a completed average of 51% of program homework. Authors concluded that the findings support program acceptability.
Wade et al, 2018 [36]	Social Participation and Navigation (SPAN) Program	Nonrandomized pilot trial^e^	15	14-22	Feasibility: Number of sessions completed, and number of social participation goals achieved during the intervention. Satisfaction: Internally developed measure to assess satisfaction with the program for participants and their parents	Feasibility: Participants completed an average of 80% of sessions (range 3-10) and achieved an average of three social participation goals (range 1-7). Authors concluded that the findings support program feasibility. Satisfaction: All participants “agreed” or “strongly agreed” that the program was useful, were glad to do the program, and would recommend the program to others. All parents “agreed” or “strongly agreed” that they were glad to do the program and would recommend it to others. Authors concluded that the findings support program satisfaction.

^a^Sample size used in the analyses.

^b^Provides feasibility outcomes for the parent study; see also the study by Wade et al [28] in Table 4.

^c^All technical problems were reported as quickly correctable without major impacts on overall sessions.

^d^Although both Recapture Life studies are based on the same parent study, a subset of 39 participants was represented in the qualitative study (McGill et al [34]) and all participants were represented in the pilot randomized controlled trial (Sansom-Daly et al [35]).

^e^Wade et al [36] is represented in Tables 3 and 4 due to reporting both feasibility and efficacy outcomes of interest.

**Table 4:** Original research publications reporting on efficacy outcomes.

Source, year	Intervention name	Study type	RCT^a^	Control group	Sample size^b^	Age range, years	Outcomes^c^
Wade et al, 2020 [28]	A Survivor’s Journey	Pilot feasibility study	No	N/A	17	13-24	Improved self-reported overall (*d*=0.58, *P*=.01) and physical quality of life (*d*=0.55, *P*<.01) at posttreatment. Improved parent-reported emotional quality of life (*d*=0.43, *P*=.03) at posttreatment.
Wade et al, 2014 [30] Wade et al, 2015 [31]	Counselor Assisted Problem-Solving (CAPS) intervention	Original RCT RCT long-term follow-up	Yes	Internet resource	132	12-17	No differences between groups in self-reported internalizing or externalizing symptoms at posttreatment. Posttreatment between groups: gains not sustained for internalizing or externalizing symptoms at 12- and 18-month follow-ups. At the 18-month follow-up: In the CAPS group, internalizing problems improved for high school–age participants only (*P*=.03).
Chadi et al, 2019 [33]	Mindful Awareness and Resilience Skills for Adolescents (MARS-A) Program	Pilot RCT	Yes	In-person MARS-A	14	13-18	No differences between groups at posttreatment in self-reported anxiety and depression. Reduced pre-post anxiety and depression for the eHealth group (Cohen *d*=0.934, *P*=.048); improvements not sustained at a 2-month follow-up. Similar frequency and duration of the mindfulness practice between groups at posttreatment.
Wade et al, 2018 [36]	Social Participation and Navigation (SPAN) Program	Pilot trial	No	N/A^d^	15	14-22	Increase in parent-reported frequency of social participation (*d*=1.11, *P*=.01), but not for teens at posttreatment. Increase in teen-reported confidence in social participation (*d*=1.45, *P*<.01) but not for parents at posttreatment. Decline in parent-reported total problems (*d*=0.96, *P*<.01), internalizing problems (*d*=0.73, *P*=.05), externalizing problems (*d*=0.79, *P*=.02), and social problems (*d*=0.82, *P*=.02), but no differences for adolescent-reported problems at posttreatment. No significant differences in the levels of social competence and confidence in the ability to manage emotions reported by teens or parents at posttreatment.
Wade et al, 2011 [37]	Teen Online Problem-Solving (TOPS) Intervention	RCT	Yes	Internet resource	35	11-18	No differences between groups in adolescent-reported parent-teen conflict, and adolescent- and parent-reported internalizing and externalizing symptoms at posttreatment. TOPS reduced parent-reported conflict compared to controls (*P*=.002) at posttreatment.
Ricketts et al, 2016 [38]	VoIP-delivered CBIT (CBIT-VoIP)	Pilot RCT	Yes	Waitlist control	20	8-16	Improved the Yale Global Tic Severity Scale score relative to controls (*d*=0.90, *P*<.01; partial η^2^=0.15, *P*<.05) at posttreatment. Higher response in the Clinical Global Impression-Improvement Scale in the treatment group (χ^2^=0.33, *P*<.05, Φ=0.41) at posttreatment. Improved Parent Tic Questionnaire score (*d*=1.38, *P*<.001; and partial η^2^=0.26, *P*<.05) at posttreatment.

^a^RCT: randomized controlled trial.

^b^Sample size used in study analyses.

^c^Statistically significant outcomes are reported with *P*<.05. *d* represents Cohen *d*. η^2^ is eta-squared. The two statistics are measures of effect size. Effect sizes are reported if information was included in the original study publication.

^d^N/A: not applicable.

### Feasibility Outcomes

Based on the seminal work of Bowen et al, there is no consensus regarding how feasibility is defined and measured [39]. Due to few published standards, guides, and thresholds upon which to test and establish feasibility of an intervention, study teams when designing feasibility studies create their own internal thresholds regarding what they determine to be important and appropriate for their specific intervention and target population. Across the five studies examining intervention feasibility included in our review, some authors used and operationalized acceptability and feasibility interchangeably, others used and operationalized acceptability and satisfaction interchangeably, and still others used and operationalized all three as distinct constructs. In Table 3, we provide detailed information on how constructs of feasibility, acceptability, and satisfaction were defined and measured by the original authors of the included studies, and their respective results. Although, as expected, we did not observe consensus across all studies in their internal thresholds, common benchmarks included enrollment, adherence, and completion rates established a priori in order to determine feasibility (ie, addressing the question of “can this be done?”) and use of internally developed measures or qualitative exit interviews to measure program acceptability and satisfaction (eg, whether participants enjoyed the program, whether participants benefited from the program, whether the program met their goals, and whether they would recommend the program to others). All studies reported high feasibility, acceptability, and satisfaction of telemental health interventions based on their own a priori internally established thresholds [29,32,34-36].

Sample sizes across studies ranged from 15 to 45. Authors reported that enrollment and program completion rates met prespecified target goals, with >30% enrollment and >70% program completion rates across studies. Patients endorsed the high benefit and low burden of participation and satisfaction with the program, and mentioned they would recommend the program to others on Likert-scale measures (ie, “agreed” or “strongly agreed” with all item measures) and/or program exit interviews. In group-based telemental health interventions, patients endorsed experiencing a sense of support, trust, rapport, and connection with the facilitator and other group members on therapeutic alliance measures and/or qualitative interviews. For the Social Participation and Navigation (SPAN) program, although patients and parents reported high levels of satisfaction and enjoyment with participation, parents were more likely than their children to report that the program was useful [36]. Across all studies, technological difficulties were reported to have a low impact on intervention delivery and treatment satisfaction. However, of note, all studies examining feasibility were conducted with participants aged 13 years or older.

### Efficacy Outcomes

There were seven efficacy studies with sample sizes ranging from 14 to 132; five had randomized designs [30,31,33,37,38] and two were prospective cohort studies [28,36] (Table 4). Of the randomized trials, three were compared to internet resource comparison groups [30,31,37], one to an in-person version of the videoconference-based intervention [33], and one to a waitlist control group [38]. Five (71%) of the efficacy studies were pilot studies with small sample sizes (ie, ≤35 participants) [28,29,33,36,38]. Four of the studies collected both patient- and parent-reported outcome measures [28,36-38]. In our review, there was a significant amount of heterogeneity among outcomes targeted by specific interventions and corresponding treatment effects. All efficacy studies had a primary focus on psychosocial/mental health outcomes. One of the prospective cohort studies also measured physical outcomes (ie, overall and physical quality of life) [28]. Outcome measures across the five randomized trial studies were variable (ie, anxiety, depression, internalizing problems, externalizing problems, behavioral symptoms, and parent-teen conflict) [30,31,33,37,38]. Treatment outcome assessment timeframes were generally assessed immediately after treatment [28,30,33,36-38]. One trial reported on outcomes at a 2-month follow-up [33], and another trial reported on outcomes at a 12-month/18-month follow-up [31].

For the two prospective cohort studies, youth and parents reported some improvements in patient mental health well-being and functioning at posttreatment [28,36]. For the first study of a cognitive behavioral problem-solving intervention (sample size=17), patients reported significant improvements in emotional, physical, and overall quality of life (medium to large effects), whereas parents reported significant improvements in patient emotional quality of life only (medium effect) [28]. For the second study of a social skills and problem-solving intervention (sample size=15), patients reported significant improvements in confidence in social participation only (large effect), whereas parents reported significant improvements in patient frequency of social participation, internalizing problems, externalizing problems, social problems, and total problems (large effects) [36].

Of the randomized trial studies, four compared the intervention of interest with an active comparison group [30,31,33,37] and one with a waitlist control [38]. In the waitlist control trial (sample size=20), a cognitive behavioral intervention was associated with significant improvements in patient- and parent-reported behavioral symptoms (large effect) [38]. In a second trial testing a problem-solving intervention compared to an active internet resource comparison group (sample size=35), there were no significant group differences in patient-reported parent-teen conflict or patient- and parent-reported internalizing and externalizing symptoms; the problem-solving intervention demonstrated significant improvements in parent-reported parent-teen conflict only [37]. In the only trial to utilize a comparison group of face-to-face delivery of the same mindfulness intervention (sample size=14), both modes of delivery resulted in improvements in patient-reported posttreatment anxiety and depression with no significant differences between groups; improvements were not sustained at a 2-month follow-up [33]. Two studies of a problem-solving intervention compared to an internet resource comparison group (sample size=132) found no differences between groups in patient-reported internalizing and externalizing symptoms at posttreatment [30] and longer-term follow-up (12-month and 18-month follow-ups) [31].

### Risk of Bias

**Figure 2:**
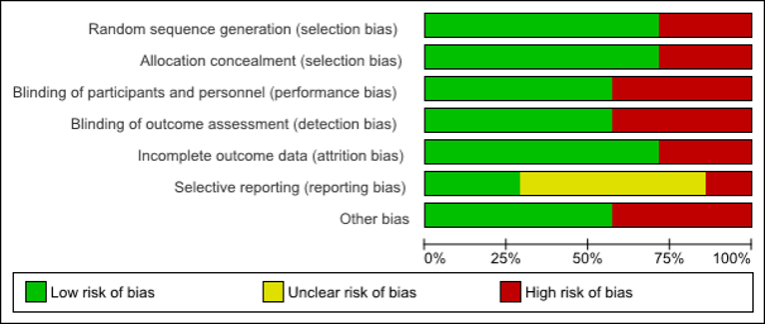
Risk of bias.

Risk of bias was evaluated for all efficacy studies (Figure 2). Of the seven studies, five reported random sequence generation and allocation concealment (ie, the randomized trials). For the blinding of participants and personnel, and blinding of outcome assessment domains, four were low risk and three were high risk; the high-risk studies consisted of study designs with no control group or a waitlist control group. For attrition bias, five were low risk and two were high risk. For selective reporting bias, two were low risk, one was high risk, and four were unclear; studies were rated as unclear due to a lack of clinical trial registration or a published protocol. For other bias, four were considered low risk and three were high risk.

## Discussion

### Principal Findings

Chronic illnesses are commonly associated with comorbid mental health problems/disorders, including anxiety, stress, depression, maladjustment, and poor coping skills [2,4]. Further exacerbating baseline disease-related stressors, the COVID-19 pandemic has perpetuated a global mental health crisis and a corresponding increase in the demand for services [11]. Telemental health has become the standard of care since March of 2020, and may provide a cost-effective, scalable, and sustainable means of remote health care delivery [40]. This is the first systematic review to summarize the research evidence in support of the feasibility and efficacy of telemental health interventions for children, adolescents, and young adults with chronic illnesses.

In this article, we identified 12 studies on telemental health interventions. The interventions focused on evidence-based treatment strategies, including mindfulness, cognitive behavioral, and problem-solving strategies, across a broad range of target illnesses and psychosocial outcomes. Five (42%) of the studies included feasibility outcomes and 7 (58%) included efficacy outcomes. All but two studies were pilot studies with relatively small sample sizes. Across the small number of identified studies, telemental health interventions seemed to be appealing and acceptable for patients and parents alike. Although navigating videoconferencing platforms did not present technological barriers to treatment attendance or engagement, it is important to note that such findings were reported for studies with teenagers and young adults only who are understandably more tech savvy than younger cohorts. Single-cohort or waitlist control pilot studies examining the efficacy of telemental health interventions showed early promise and medium to large treatment effects. In randomized trials with active comparison conditions, there was little evidence of significant treatment effects across a range of mental health symptoms, and few studies included long-term outcome assessments. Only one trial compared face-to-face and telemental health delivery of the same intervention; both modes of delivery were similarly efficacious and improvements were not sustained at longer-term follow-up.

Together, this set of preliminary studies examining feasibility and efficacy provides early evidence that (1) telemental health interventions may be appropriate and acceptable to patients and their parents; (2) videoconferencing platforms may not present technological barriers to engagement and use; and (3) there is some modest early evidence in support of the efficacy of telemental health interventions, but preliminary findings are mixed. Our findings are consistent with previous reviews suggesting that telehealth may be appropriate and efficacious for adults with chronic conditions and for the delivery of mental health care [41,42].

### Limitations

Some limitations need to be considered. First, relatively few papers have been published on telemental health feasibility and efficacy among children, adolescents, and young adults with chronic illnesses. This suggests that the science lags behind its rapid rate of adoption in clinical settings. Second, most papers were pilot studies with small sample sizes and were underpowered to detect clinically or statistically significant treatment effects. Third, given the heterogeneity of treatment type, disease target, measurement timepoints, and mental health outcome measures, we were unable to perform a meta-analysis to quantify treatment effects. Fourth, approximately one-third of studies did not report on the racial or ethnic distribution of their sample, and those that did reported that the majority of participants were white. Similarly, none of the studies reported on the rurality of their sample. Fifth, we limited our study to English-language publications. Thus, the generalizability of the findings remains unclear. Sixth, it was not possible to examine age and developmental differences in treatment effects due to the small number of included studies and specific interventions developed and tested with a wide age range of patients. This limitation is consistent with a previous review that found little research assessing age and developmental patterns in coping with chronic illnesses [6]. Finally, our study was limited to peer-reviewed published articles and did not consider unpublished gray literature, such as conference abstracts and dissertations, which may have led to the identification of additional studies.

### Future Directions

Future research should extend beyond feasibility and early efficacy pilot studies to assess how telemental health delivery compares to in-person care in therapeutic alliance and rapport building, treatment engagement and treatment drop-out rates, and efficacy/effectiveness in large-scale randomized trials. Although some preliminary evidence suggests positive effects associated with telehealth delivery, it is important to examine the relative benefits and costs associated with remote interventions. Telehealth may not be an appropriate delivery format for all patients. Some patients may respond better to or prefer in-person care, experience higher homework compliance, have lower dropout rates, and/or establish a stronger therapeutic alliance with a provider in a face-to-face meeting. Important future research directions include the development of the best screening processes to match patient characteristics to care delivery preferences in order to optimize outcomes. Patient characteristics with the potential to impact care delivery that warrant further exploration include age and developmental stage, acuity of mental health needs, chronic illness diagnosis and illness narrative, and medical treatment.

Evidence-based strategies proven to be effective when deployed through traditional in-person care may require iterative intervention adaptations to successfully translate treatment effects to telehealth modes of delivery. It may not be as straightforward as simply delivering the same manualized protocols via Skype, Zoom, or WebEx, and the optimal balance between traditional face-to-face care and remote delivery should be further examined. Equity in access to telemental health should also be examined, as some research teams supplied videoconferencing capable devices and high-speed internet access to participants who needed them, and such an approach may not be scalable as the standard of care. Ultimately, this study and future studies will help inform whether, for whom, and under what treatment conditions telemental health has the potential to serve as a sustainable long-term alternative to in-person care after the COVID-19 pandemic.

### Conclusions

The strengths of this paper include the systematic approach to synthesizing the breadth of literature across chronic illness populations and the timely focus on telehealth, which has displaced in-person treatment as the standard of care in the context of the COVID-19 pandemic. Our findings suggest that although COVID-19 has necessitated remote treatment delivery, and patients and families may find this mode of delivery to be engaging and satisfactory, the state of the science is in a nascent stage and there is much to be learned about whether such interventions work, for whom they work, and in what contexts they work, as well as how they compare to in-person treatments.
